# Tuberculous aortitis as a rare cause of aortobronchial fistula with massive haemoptysis: A case report

**DOI:** 10.1016/j.ijscr.2020.04.078

**Published:** 2020-05-08

**Authors:** Joseph Motshedi Sekgololo, Chauke Risenga Frank, Vally Moinuddeen, Dehghan-Dehnavi Alireza, Khaba Moshawa Calvin

**Affiliations:** a Cardiothoracic Surgery Department, Sefako Makgatho Health Sciences University, Dr George Mukhari Academic Hospital, Pretoria, South Africa; b Vascular Surgery Department, Sefako Makgatho Health Sciences University, Dr George Mukhari Academic Hospital, Pretoria, South Africa; c Radiology Department, Sefako Makgatho Health Sciences University, Dr George Mukhari Academic Hospital, Pretoria, South Africa; d Anatomical Pathology Department, Sefako Makgatho Health Sciences University, Dr George Mukhari Tertiary Laboratory, National Health Laboratory Service, Pretoria, South Africa

**Keywords:** Aortobronchial, Aortopulmonary, Aortobronchopulmonary, Fistula

## Abstract

**Background:**

Aortobronchial fistula is a rare condition, which is difficult to diagnose. It is fatal if misdiagnosed or not well treated. Massive haemoptysis is usually the first common symptom. Computed tomography angiogram (CTA) is the best non-invasive diagnostic modality. Treatment options include open repair procedure or Transthoracic Endovascular Aortic Repair (TEVAR) and resection of the destroyed lung tissue. The recurrent rate is high.

**Case presentation:**

This report is a case of a 26-year-old African female patient who presented with massive haemoptysis. She had been treated for pulmonary tuberculosis two years before. The patient was diagnosed with retroviral disease and had been on treatment for two years. She underwent a 2-stage repair procedure. The initial treatment was TEVAR, which was followed by lung resection after two weeks. Both operations were uneventful. Histopathology analysis confirmed tuberculous aortitis as aetiology. The patient had been followed up for a year, with no recurrence.

**Discussion:**

Aortobronchial is divided into primary and secondary subtypes. Primary aortobronchial fistula is commonly caused by inflammatory disease and atherosclerosis. Secondary aortobronchial fistula is a complication of surgery for thoracic aorta and congenital cardiac disease. Tuberculous fistula is an uncommon cause of aortobronchial fistula. Surgery for aortobronchial fistula should include controlling both aortic and pulmonary fistula sites. A healthy tissue or muscle flap should be used between the repaired sites to prevent refistulisation. Recurrence is common; hence, long-term follow up is important.

**Conclusion:**

Early diagnosis and adequate treatment are important. A high index of suspicion is important for diagnosis, because the diagnosis is difficult.

## Highlights

• It is a rare condition that is invariably fatal if not diagnosed or treated on time.
• Aortobronchial fistula is subdivided into primary and secondary fistulae.
• Diagnosis of aortobronchial fistula requires high index of suspicion.
• Long term follow up is important because of significant recurrent rate.

## 1 Background

Aortobronchial fistula is a rare condition that is invariably fatal if not diagnosed and treated well [1]. The common causes include: aneurysms and endovascular stents/infected thoracic aortic graft. However, the sequela of pulmonary tuberculosis in a form of necrotizing pneumonia has been implicated. Tuberculosis is an uncommon cause of aortobronchial fistula; especially, if primary pathology is of cardiovascular in origin (tuberculous aortitis), which is the case in this report [2]. With appropriate surgical intervention, survival rate greater than 70% can be achieved [1].

## 2 Case report

**Fig. 1:**
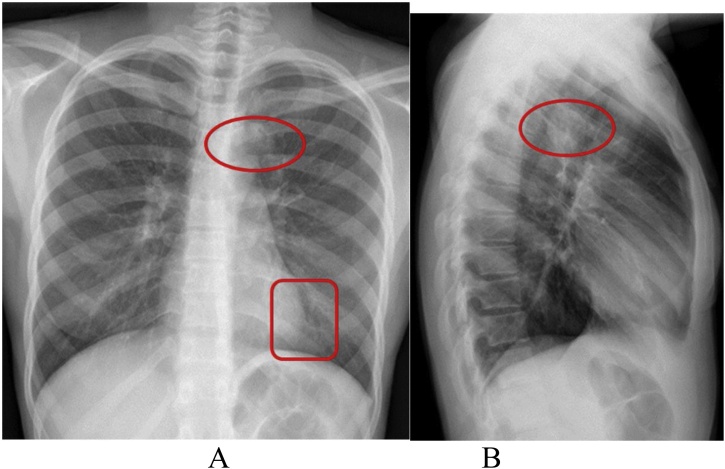
A, B: PA and lateral chest X-Ray: Left upper lobe apicoposterior segment opacity obscuring aortic knob (shown by ). Left lower zone opacity obscuring left cardiac border (shown by ).

**Fig. 2:**
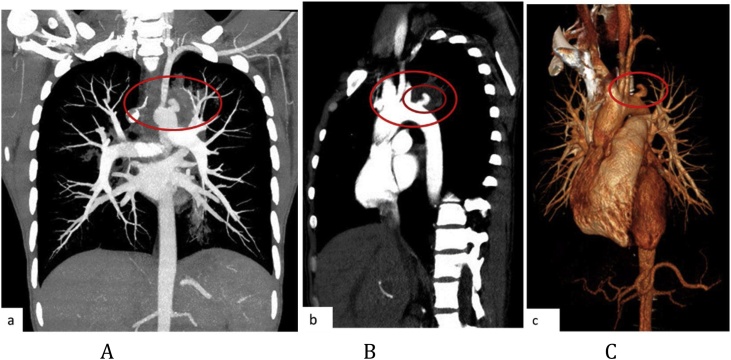
A: CT angiogram of the chest, MIP coronal. B: MIP parasagittal. C: Coronal reconstructed. There is outpouching contrast content reverse ‘S’ shape structure of the aorta lateral to the left subclavian artery with peripheral density as shown by ().

**Fig. 3:**
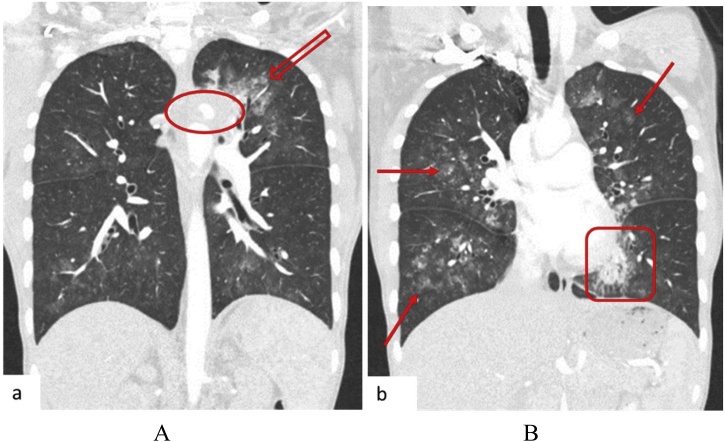
A CT angiogram of the chest, lung window showing left upper lobe pleural density adjacent to the aortic knob () with surrounding lung density (). B: Bilateral diffuse patchy scattering lung opacities mostly centrilobular () and left lingula inferior segment consolidation ().

**Fig. 4:**
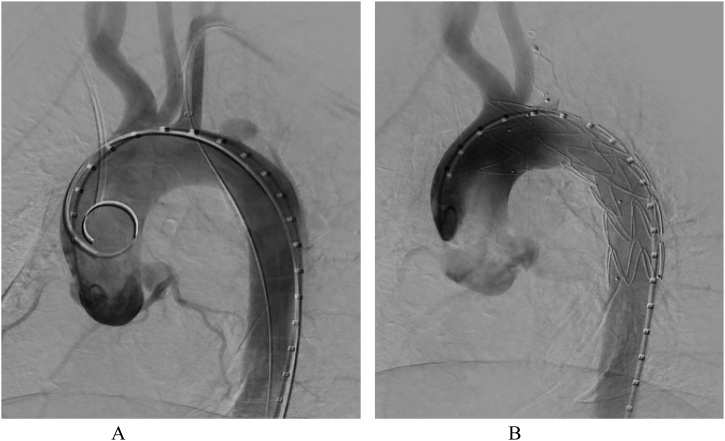
A: Angiogram of thoracic aorta, showing bovine trunk and focal aneurysm distal to LSA. B: Stentgraft is deployed, resulting in complete occlusion of LSA. Vascular plug (Amplatzer) was deployed at the origin of LSA to prevent endoleak.

**Fig. 5:**
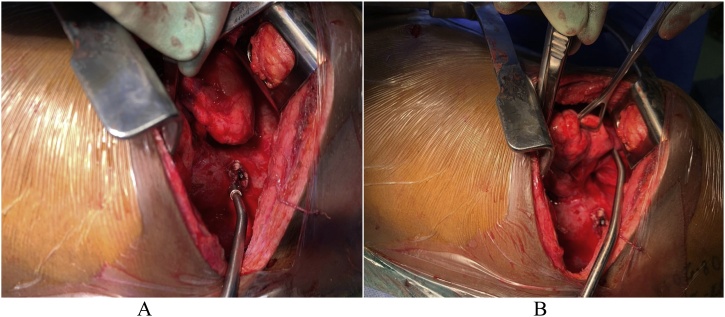
A: Through posterolateral thoracotomy, left apico-posterior segment was mobilized from aorta, exposing aortic fistula site as pointed out by the end of the Yankauer suction tip. B: An apico-posterior segment was retracted anteroinferiorly to expose the pulmonary site of the fistula.

**Fig. 6:**
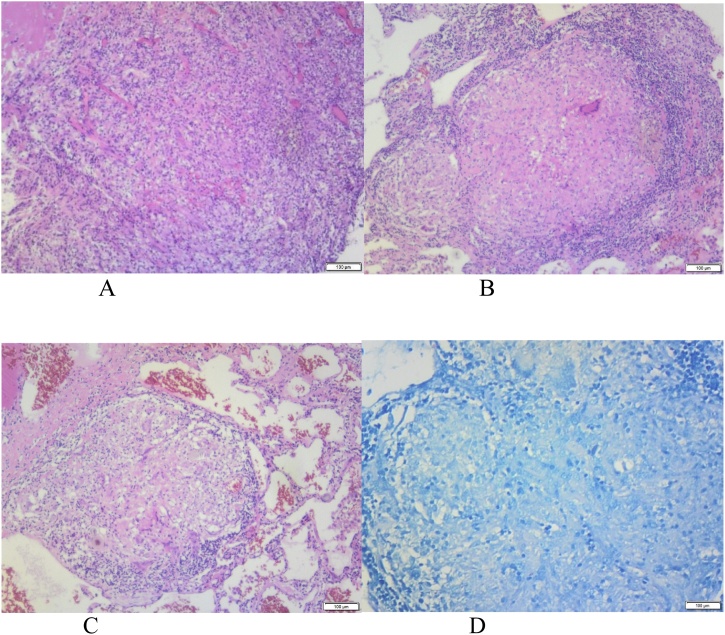
A: Poorly formed granuloma with admixed chronic inflammation. B: Well formed granuloma with Langhan-type giant cell and central necrosis. C: Granuloma with admixed Langhan-type giant cell. D: Negative Ziehl Neelsen (ZN) stain for fast bacilli.

In compliance with SCARE criteria (Agha et al. [3]), this report is a case of a 26-year-old African female, who presented with massive haemoptysis, chest pain and persistent cough. The patient had been treated for pulmonary TB two years before and had Human Immunodeficiency Virus, which was being treated with antiretroviral drugs. The patient was acute-on-chronic ill looking and clinically wasted. She had mild respiratory distress with respiratory rate of 22 breaths per minute. Other vital signs were normal. Physical examination revealed pale sclera and palms, which were suggestive of anaemia. On chest examination, there were crepitations on the left upper lobe area with no murmurs audible to suggest the presence of the fistula. The baseline blood confirmed anaemia with haemoglobin of 5.1 g/dl and other bloodlines were normal. An International normalized ratio (INR), urea, electrolytes and creatinine were also normal. Resuscitation was undertaken while completing other investigations. Chest X-ray (CXR) was done, with posteroanterior (PA) and lateral views showing left upper zone opacification obscuring the left cardiac knob [Fig. 1A,B]. The left lower zone had an opacity obscuring the left cardiac border, suggestive of multilobar infection/vascular pathology. In subsequent computed tomography angiogram (CTA), there was a reverse “S” shaped outpouching contrast content structure from aortic arch lateral to the Left Subclavian Artery (LSA) origin with adjacent pleural density and surrounding the lung opacity. The features were suggestive of aneurysm, which complicated with aortobronchial fistula surrounded by haematoma [Fig. 2A–C]. The lingula segment of the left upper lobe had consolidative changes and there were also bilateral diffuse patchy scattering centrilobar opacities, which suggested an active pulmonary infection [Fig. 3A,B]. The patient was admitted to Intensive Care Unit (ICU) for optimization in preparation for surgery. Medical therapy in the form of antitussive, sedatives, antifibrinolytics and empirical antibiotics were provided. The patient did not require any security of the airway or mechanical ventilatory support. The patient underwent TEVAR two days after ICU admission. The procedure was done via general anaesthesia with invasive lines monitoring. The right femoral artery was exposed via oblique groin incision. The left common femoral artery was punctured with 5Fr sheath for angiographic access. The initial angiogram showed that the Left Common Carotid Artery (LCCA) had a common origin with brachiocephalic trunk [Fig. 4A]. At approximately 10 mm distal to the LSA, a focal aneurysm was noted on the proximal descending aorta, with 13 mm base. The appearance suggested a vascular aneurysm (Fig. 4A). There was no opacification of the pulmonary vasculature. Through the brachial artery approach, a vascular occlusion plug (Amplatzer) was deployed to the origin of the LSA to minimize the risk of the endoleaks. The TEVAR was performed via the right groin access, using Medtronic navion thoracic stentgraft with coverage of the LSA [Fig. 4B]. The completion angiography showed appropriate opacification of brachiocephalic trunk and its branches, including the LCCA. There was no flow into LSA. Fistula and aneurysm were excluded. Postoperative phase was uneventful. Within the same admission (after two weeks) the patient was taken for second stage of the operation, which involved apical-lower segmentectomy and apace-posterior segment debridement and pulmonary fistula repair. The approach was via muscle sparing posterolateral thoracotomy with the chest entered through the 5th intercostal space, over unresected 6th rib. Intra-pleural mobilization of the lung was undertaken with the use of both blunt and sharp dissections. A haematoma was identified within apical-lower segment. The apical part of apico-posterior segment was attached to the aorta at the fistula site, which on mobilization the two were separated without bleeding [Fig. 5]. The fistulae site on both aorta and lung were biopsied for microbiological and histopathological studies. An oblique fissure was complete, with less dense adhesions, for which adhesionolysis was done. The interlobar artery was identified and its overlying pleura was opened and dissected. The apical lower segment artery was identified, dissected, suture ligated and divided. The vein draining the apical-lower segment was also dissected and controlled in similar manner. The bronchus to apical lower segment was dissected and clamped, and an anaesthetist was asked to insufflate the lung for delineation of segment. The GIA stapler was used to resect apical lower segment bronchus. The stump was immersed into saline and was not leaking. The apical part of apico-posterior segment adjacent to area of the fistula was necrotic. It was debrided and closed with continuous Prolene 4/0 suture without using a healthy or pleural tissue for buttressing, because the tract was too small and was thoroughly debrided. A tissue of the fistula stump on the aorta was sampled for microbiological and histopathological studies. The aortic fistula stump was approximated with interrupted Prolene 4/0 suture. A size 7.5 rigid bronchoscope was passed into the airway, the trachea and the carina looked normal. No fistula was visualized within airway. The operation was uneventful and the patient was discharged and reviewed after two weeks for general follow up and histopathology results. Hematoxylin and Eosin (H&E) sections [Fig. 6A,B] showed fibrous connective tissue with poorly formed granulomas while lung sections showed necrotizing granulomatous inflammation. The granulomas were composed of aggregates of epitheliod histiocytes with central caseating necrosis and Langhan-type giant cells. Acid-fast fast bacilli or fungi were not evident. Although acid-fast bacilli were not identified [Fig. 5C,D], mycobacterial infection was still favoured. Correlation with microbiological culture and susceptibility testing was advised. The patient was put on anti-tuberculous treatment for nine months, which she completed uneventfully. She is currently coming for follow-ups every three months to check for possible recurrence.

## 3 Discussion

Aortobronchial fistula is also referred as aortopulmonary/aortobronchopulmonary fistula. It is communication between the aorta and bronchial tree. The first case of aortobronchial fistula was described in 1934 [4]. The aortobronchial fistula is divided into two subtypes, which are primary and secondary. While primary aortobronchial fistula is caused by primary pathology of the aorta or the lung, secondary aortobronchial fistula is due to postoperative complications such as erosion of bronchial tree by stentgraft and localized sepsis resulting from infected aortic replacement by the graft. Most aortopulmonary fistulae originate from thoracic aortic aneurysms, which cause erosion of pulmonary parenchyma or bronchial tree [4]. The Primary aortobronchial fistula causes include: tuberculosis (TB), syphilis, fungal infections, trauma and atherosclerosis [4]. TB and syphilis, which resulted in aortobronchial fistula, were the leading causes of mycotic aneurysms before 1960 [1]. Secondary aortopulmonary fistulae are due to surgical corrections of congenital anomalies such as aortic coarctation, which requires the graft interposition and TEVAR. The causative mechanism of aortobronchial fistulae includes compression by aortic aneurysm resulting with pressure necrosis [1]. The site of fistula is determined by anatomical position. The thoracic descending aorta and the left bronchial tree are common site of the fistula. The right-sided aortobronchial fistulae are uncommon, and if they occur, the likely cause is tuberculosis. Most patients present with episodic massive haemoptysis. However, some would have sentinel bleed which stops due to clot formation. Once the clot is lysed, a massive haemoptysis results. High index of suspicion is needed for the patient with first episode of massive haemoptysis with history of thoracic aortic surgery or congenital cardiac anomaly correction. The other compressive symptoms secondary to aneurysm include: dyspnea, cough and chest pain. The preoperative diagnosis of aortobronchial fistula is difficult. A CXR maybe normal/have alveolar infiltrates that are confined to the lobe/the segment involved. CTA is good at identifying aneurysm, but has a poor fistula visualization accuracy of 17% [4]. It also helps in diagnosing haematoma, mural thrombus and contrast leakage into the pulmonary parenchymal [4]. Bronchoscopy as the first diagnostic modality maybe catastrophic due to exsanguination. This diagnostic tool is the best modality in identifying the source of bleeding [1]. Treatment principles include: the repair/replacement of fistula or aorta, debridement and repair of bronchial opening. The wedge/lobectomy of affected lung tissue maybe required. It is advisable to use healthy tissues such as pleura/muscle between repaired areas to avoid the recurrent fistula. The procedure may be single/2-stage procedure. Two-stage procedure involves controlling fistula by using stentgraft, which is followed by lung resection after 2–3 weeks. The 2-stage procedure is undertaken in hybrid theatre where TEVAR is followed by thoracotomy. The open procedure for both aortic replacement and lung tissue resection can be done in the conventional theatres. The treatment should be individualized because an unstable patient will require resuscitation in ICU, which, is followed by open surgery/TEVAR. Open surgery may be conducted with the use of partial/total cardiopulmonary bypass (CPB). Traditionally, surgeries involved aortic fistula site correction with patch closure or prosthetic graft replacement, or (less often) by direct suturing [5]. The operative mortality of open aortobronchial fistula repair ranges from 15 to 41% [6]. The reasons for high mortality rate in the open aortic repair/replacement procedure are attributed to the use of aortic cross clamp and CPB. TEVAR has shown favourable perioperative outcomes: 30-day mortality rate of 5.9% and no cases of paraplegia [7]. The total recurrent rate of aortobronchial fistula is 11.1% [7].

## 4 Conclusion

Aortobronchial fistula is rare condition, which requires high index of suspicion for diagnosis. A massive haemoptysis is common and usually a first symptom. Tuberculous aortitis is an extremely unusual form of cardiovascular tuberculosis resulting in aortopulmonary fistula. CTA is the best non-invasive diagnostic test. Treatment involves controlling both aortic and bronchial fistula ends. Due to high recurrence rate, long-term follow up is recommended.

## Declaration of Competing Interest

None.

## Sources of funding

None.

## Ethical approval

Sefako Makgatho University Research Ethics Committee (SMUREC) approved the publication of this case report.

INSTITUTIONAL REVIEW BOARD (SMUREC/M/29/2020).

## Consent

Written informed consent to publish this case report and images was obtained from the patient. The copy of written informed consent will be available for Editor-in- Chief on request.

## Author contribution

All authors wrote the case report.

Dr. Sekgololo J.M organized the manuscript and critically revised the paper.

## Registration of research studies

Not applicable, because is a single case report.

## Guarantor

Dr. Sekgololo J.M.

## Provenance and peer review

Not commissioned, externally peer-reviewed.
